# Reducing occupational stress with a B-vitamin focussed intervention: a randomized clinical trial: study protocol

**DOI:** 10.1186/1475-2891-13-122

**Published:** 2014-12-22

**Authors:** Con Stough, Tamara Simpson, Justine Lomas, Grace McPhee, Clare Billings, Stephen Myers, Chris Oliver, Luke A Downey

**Affiliations:** Centre for Human Psychopharmacology, Swinburne University, PO Box 218, HawthornVictoria, Melbourne, Australia; NatMed Research Unit, Southern Cross University, Lismore, NSW Australia; Department of Psychology, Swansea University, Swansea, Wales UK

**Keywords:** B vitamins, Stress, Cognition, Genetics, Homocysteine, Occupational stress, Work-related stress, MRI, fMRI, MRS

## Abstract

**Background:**

Workplace stress in Australia and other western countries has been steadily increasing over the past decade. It can be observed not only in terms of increased compensation claims but also costs due to absenteeism, loss of productivity at work and reduced psychological and physiological health and well-being. Given the cost and pervasive effects of stress in the modern workforce, time efficient and cost-effective interventions capable of reducing occupational stress (or strain) and burnout are urgently required for the improved well-being of stressed employees. One intervention gaining scientific traction is supplementation with nutritional interventions, particularly the B group vitamins.

**Methods:**

This study was developed to examine the effects of B group vitamins on workplace stress and mood variables with a sample of full-time employed older adults who subjectively report feeling stressed. The study is a randomized, double-blind, placebo-controlled, parallel-groups clinical trial where 200 (N = 100/group) participants will be randomized to receive Blackmores® Executive B Stress Formula or placebo daily for a period of 6 months. Participants will be tested at baseline and 6 months post-randomization on workplace stress, cognitive, personality and mood measures, cardiovascular (brachial and aortic systolic and diastolic blood pressures as well as arterial stiffness), biochemical (assays to measure inflammation and safety) as well as genetic assessments (to assess stress processing) and neuroimaging measures (to investigate *in vivo* mechanisms of action of B vitamins). In addition to this pre- and post- supplementation testing, participants will also complete a battery of self-report questionnaires online to assess their stress and mood once a month for the duration of the study. The primary aim of the study is to investigate the effects of B vitamin supplementation on work related stress. The secondary aims are to explore the mechanisms underpinning any changes in mood or workplace stress due to the B vitamin intervention by examining relationships between cognitive, biological, neuroimaging and cardiovascular variables over 6 months. A subset of 40 participants (N = 20/group) will undergo neuroimaging at baseline and at 6 months using functional magnetic resonance imaging (fMRI) and magnetic resonance spectroscopy (MRS) in order to further explore *in vivo* mechanisms of action of B vitamins.

**Trial registration:**

Australia and New Zealand Clinical Trials Register (ANZCTR):ACTRN12613000294752

## Background and rationale

Stress in the workplace is an increasing problem in Australia and most Western countries. Stress related claims have steadily risen each year in all states of Australia from 1997 to 2006 [1]. Additionally, it has been estimated that occupational stress costs the Australian economy $14.81 billion per year in stress related workplace inactivity and absenteeism at work [2]. The World Health Organization (WHO) has stated that the direct cost of occupational stress in 1992 in the USA was US$42 billion [3]. The cost of high levels of Occupational Stress can be seen at personal, organizational and societal levels. Workers who experience high occupational stress are significantly more likely to make a stress-related work cover claim, use significantly more medical resources and contribute less to an organization through increased absenteeism and lost productivity. Prolonged experience of high levels of stress and not being able to meet workplace demands can lead to ‘burnout’. Workers experiencing greater mental and physical health issues are costly to treat whether the symptoms are chronic or acute, reducing the worker’s ability to return to the workplace. The WHO has claimed that in the USA some US$300 billion is lost in productivity, absenteeism and staff turn-over each year due to high levels of occupational stress (amounting to approximately 2.6% of US GDP). These data highlight the need for time and cost effective interventions to reduce occupational stress.

## What is occupational stress?

The US National Institute for Occupational Health and Safety (NIOSH) has argued that Occupational stress occurs when there is a discrepancy between the demands of the environment/workplace and an individual’s ability to carry out and complete these demands. Increased workplace demands diminish the ability of our body to deal with stress [4]. Additionally, poorer diets because of longer hours spent at work also result in a diminished capacity for our body to deal with stress.

## Dietary vitamins in the central nervous system

The central nervous system is dependent upon the nutrients supplied through a good, varied, healthy diet. Good nutrition is imperative, particularly for maintaining the brain’s structure and normal cognitive function [5]. Vitamins and micronutrients play a significant biochemical role in maintaining cognitive processes within the brain. The role of antioxidants and B vitamins contained in food and their relationship to maintaining cognitive processes and general health is discussed below.

i. *Antioxidants*

Fresh fruits, green vegetables and berries contain a rich source of vitamins A, C and E. These food sources contain dietary antioxidants that can prevent, inhibit or repair damage caused by oxidative stress [5, 6]. Oxidative stress is representative of an imbalance or disruption in the redox state (oxidation/reduction reactions) within cells causing impaired signalling and regulation, resulting in impaired functioning [7]. The maintenance of redox homeostasis is essential for healthy cellular function. Antioxidant vitamin C levels are particularly high in the brain and are needed for the transformation of dopamine into noradrenalin as well as the production of some neurotransmitters [5]. Vitamin E is a lipid soluble antioxidant which has neuroprotective effects against free radical damage, preventing cellular injuries to the brain related to oxidative stress [8]. Vitamins E and A protect against lipid peroxidation, a damaging process affecting the permeability of cell membranes [9]. Vitamin C works synergistically with B group vitamins and is vital for the metabolism and utilization of folate/folic acid [10].

i. *B Vitamins*

B group vitamins can be found in a wide range of foods like whole grains, bananas, beans and meats. Folate/folic acid, B6 and B12 represent some of the B group vitamins. Folate, B6 and B12 vitamins are known to contribute to the regulation of healthy levels of the amino acid HCy, and share a synergistic role in the maintenance of cardiovascular and neural health, and are also vital for energy metabolism [5, 8, 9]. Cross sectional and prospective studies have identified that elevated plasma HCy levels is associated with the promotion of spontaneous cell death (apoptosis), incidence of stroke, brain atrophy, Alzheimer’s disease, bone fracture and is considered a risk factor for cerebrovascular disease [11, 12].

Folate/folic acid, B6 and B12 vitamins are essential for the methylation of HCy to methionine in the central nervous system. Methionine plays a crucial role in one-carbon metabolism; biological processes for DNA synthesis, repair and other methylation reactions [13]. If HCy is not sufficiently converted back to methionine, the methylation process will be inhibited resulting in a build-up of HCy. An elevated level of HCy increases the likelihood for oxidative stress, leading to negative events like mitochondrial membrane damage and DNA strand breakage [14, 15]. The role of HCy in disease pathogenesis remains unclear. HCy could play a direct role in the disease process or be simply a marker of folate, B6 and or B12 vitamin deficiency. However, research has identified that chronic stress depletes vitamin B6 [16] while supplementation with B6 vitamins could be a therapeutic strategy in reducing stress [17]. Therefore, one mechanism by which B group vitamin intervention may aid the reduction of stress and fatigue ratings of those in full time employment is through the uninhibited clearance of HCy.

In addition, neuroimaging studies suggest that high levels of plasma HCy and low levels of B vitamins are linked to a higher incidence of brain atrophy and degeneration [18, 19]. This type of data suggests that HCy plays a role in brain ageing, by contributing to subclinical brain changes in what we would believe to be an otherwise healthy population.

## Vitamin supplementation interventions for work stress

One potential pharmacological intervention to assist our bodies to cope with increased levels of stress that has received some preliminary support recently is vitamin B supplementation. Dietary deficiencies in micronutrients have been implicated in altered mood states (including work stress and psychiatric symptoms) in otherwise healthy individuals [20]. Supplementation with micronutrients to overcome these dietary deficiencies has been observed to improve perceived stress, mild psychiatric symptoms, and some aspects of everyday mood in a recent meta-analysis of studies examining short-term multivitamin supplementation [21]. These positive effects have been postulated to have occurred through alteration of biochemical processes affected by poorer dietary status through supplementation. Specifically, high doses of B vitamins have been suggested to be effective in improving mood states in both clinical and non-clinical populations.

## Previous studies examining the relationship between B vitamins and stress

Work stress is the result of an imbalance between the assessment of environmental demands and an individual’s resources and skills [22]. Despite the widespread use of vitamins to compensate for the busy lifestyle and irregular eating patterns that often accompany stress, there are few controlled trials directly investigating the relationship between multivitamins and psychological strain [23]. Despite strong advertising campaigns from many Vitamin companies, many of the claims do not have strong evidence. Therefore more studies are urgently required to help inform the public about the efficacy of vitamins on states such as stress which are often targeted by vitamin companies. There are a few smaller studies on this topic. Two studies using a multivitamin formula observed a reduction in stress symptoms after supplementation of just 28 and 30 days [24, 25]. Schlebusch and colleagues used a well-designed protocol, and screened for a highly stressed sample [25]. After 30 days of supplementation, significant treatment effects were evident, with the multivitamin reducing the level of anxiety and stress and improving psychological well-being. Carroll and colleagues [24] used a similar design with a smaller sample but employed more stringent exclusion criterion. Eighty male participants were supplemented over 28 days. They demonstrated significant reductions in anxiety and perceived stress in the multivitamin group in comparison to the placebo group. These participants also reported being less tired and having greater concentration compared to the placebo group. Our research centre has recently examined whether a popular multivitamin supplement available in Australia, (Blackmore’s Executive B Stress Formula) containing a complex of mostly B group vitamins, improved work related stress (the only study to address work stress variables) [23]. The duration of administration of the multivitamin in this study was 90 days, a significant increase in comparison to previous studies of 30 days of multivitamin administration [24–26]. Sixty participants, recruited from the community, completed the 3-month, double-blind, randomized, placebo-controlled trial in which personality [27], work demands, mood, anxiety and strain were assessed. The primary analysis revealed that the Vitamin B group reported significant reduction in *Personal Strain* (p = 0.02), from weeks 4 (M 92.10, SE 2.44) to week 12 (M 85.54, SE 2.27), while the placebo group showed a significant increase in levels of strain from week 4 (M 88.84, SE 4.33) to week 12 (M 93.44, SE 4.03).

## B vitamins and cognition

Cross sectional and prospective studies in the elderly have revealed a positive relationship between cognitive performance and B vitamin intake, and a negative relationship between cognitive performance and B vitamin deficiency as evidenced through plasma HCy levels [28]. It is through the process of methylation reactions that B Vitamins are believed to influence cognitive performance [29]. It has therefore been postulated that the HCy lowering properties of B vitamins, could potentially mitigate the effects of cognitive decline [30, 31].

Recently, studies have emerged that have identified a link between brain atrophy, cognitive ability and B vitamin levels. For example, Smith and colleagues [32] investigated vitamin B6, B12 and folic acid supplementation with an older sample that were experiencing mild cognitive impairment, and observed that the rate of brain atrophy declined in those who consumed vitamins compared to placebo. They also observed that high brain atrophy was associated with lower cognitive test scores. Similar results were obtained by de Jager and colleagues [33], where vitamin B supplementation significantly slowed cognitive impairment over 2 years. This was especially the case in those who had high levels of plasma HCy. Promising results have also been detected with folic acid supplementation alone, where 3 years of supplementation significantly improved cognitive facets relating to processing speed, memory and attention [28]. It must be noted, however, that these studies have focussed on participants who are elderly and therefore experience cognitive decline more readily than younger individuals. It also highlights the need for better vitamin B nutrition in those who are older and working, where cognitive decline is both prevalent and occupationally detrimental.

Few randomised controlled trials have investigated the cognitive benefits of vitamin intake in younger, healthy cohorts. However, a randomized controlled study conducted by Pipingas and colleagues [34] investigated the effects of 16 weeks supplementation of a multivitamin with 138 participants aged between 20 and 50 years. While these researchers did not observe any significant improvements in cognitive ability, they noted that plasma folate, B6 and B12 levels were increased and HCy levels decreased after multivitamin supplementation.

## The contribution of B vitamins to cardiovascular and neural health

Healthy vitamin and mineral status is paramount for healthy neurocognitive function and well-being [35]. During pregnancy it is an accepted practice for women to supplement their diet with folate. Early studies established that dietary supplementation of folic acid reduced the risk of babies being born with neural tube defects such as spina bifida and anencephaly [36]. Folic acid intake is therefore paramount during pregnancy to assist the proper closure of the foetal neural plate which under rapid development forms the spinal cord and skull.

A wide variety of neurological symptoms manifest in humans as a result of vitamin B12 deficiency. Numbness of the skin, hands or feet, muscle weakness and disjointed motor coordination are some of the symptoms indicative of sensory and motor disorders as a result of the degeneration of the spinal cord due to inadequate myelin [37]. Whereas confusion, apathy, depression and dementia like symptoms are indicative of cerebral disorders that can vary in severity and can include memory and judgement impairments [37]. When folate or vitamin B12 is insufficient, an accumulation of HCy occurs which is understood to be highly toxic to neurons and believed to be the cause of these neurological, sensory and motor symptoms described [38]. Therefore, it is suggested that consumption of vitamin B12 will address the accumulation of HCy, and in turn, alleviate the associated neurological, sensory and motor system symptoms.

Folate, B6 and B12 vitamins are the key vitamins that have a direct effect on mood and neurotransmitter regulation. Methionine is required in the synthesis of S-adenosylmethionine (SAM), solely responsible for methylation reactions in the brain [39]. The products of these reactions include the neurotransmitters related to psychological well-being such as dopamine, serotonin and norepinephrine in addition to phospholipids, proteins, DNA and myelin [29]. Furthermore, SAM is essential for the maintenance of choline as well as the production of acetylcholine in the central nervous system (important for memory and mood, skeletal muscle control, heart rate and breathing and transforming cysteine into the most abundant antioxidant in the body, glutathione) [40]. Research has established the efficacy of SAM as an anti-depressant treatment for those suffering from depression with recent reviews detailing that supplementation with SAM for four weeks or more is as effective as treatment with tricyclic antidepressants for clinical depression [41, 42]. In terms of cognitive effects, B12 vitamin and folate deficiency may cause a disruption to the SAM pathway, leading to a reduction in the production of neurotransmitters. Therefore, deficiency may have a direct effect on mood and cognitive function by virtue of these vitamins in the production of neurotransmitters [43].

A clear association exists between HCy levels and cardiovascular health [14]. An increased level of HCy has a harmful effect on the cardiovascular system through actions that promote blood clotting with platelets and the release of growth factors for vascular endothelium [44]. A reduction in the bioavailability of a powerful vasodilator, endothelial nitric oxide, may be related to vascular damage [38]. Kang and colleagues [45] reported a relationship between job-related stress, plasma HCy levels and cardiovascular risk factors in a group of 152 workers. Similarly, other researchers [17] reported a significant relationship between acute psychological stress and elevated plasma HCy levels, blood pressure and heart rate. They concluded that one mechanism, high HCy, through psychological stress may contribute to the initiation and progression of vascular disease. These findings suggest that the interrelationship between B vitamins and folate in the regulation of HCy may affect cognitive function via direct effects on the brain or indirectly via mechanisms working on the cardiovascular system. As such, examination of the effect of B-vitamin supplementation upon stress, mood, and cognitive performance alongside measurement of probable biological and neurological mechanisms of action of B vitamins should help clarify the therapeutic role of these commonly available supplements.

## Study protocol: reducing occupational stress with a B-vitamin focussed intervention

Given the growing incidence, cost and pervasive effects of stress in the modern workforce, the primary objective of this research is to determine whether administration of B-vitamins reduces occupational stress (or strain), burnout, and the cost of absenteeism in a population of older workers with high levels of occupational stress. We will examine the relationship between B-vitamin supplementation, workplace stress, cognitive, personality and mood measures, cardiovascular (brachial and aortic systolic and diastolic blood pressures as well as arterial stiffness), biochemical (assays to measure inflammation and safety) as well as genetic assessments (to assess stress processing) and neuroimaging measures (to investigate *in vivo* mechanisms of action of B vitamins) to identify the mechanisms through which B-vitamin supplementation may improve the well-being of Australian workers.

## Design and methodology

### Design

This study is randomized, double-blind and placebo-controlled, 2-arm parallel-groups clinical trial with participants to receive Blackmores® Executive B Stress Formula or placebo.

### Aims and study hypotheses

The primary aim of this study is to investigate 6 month effects of a B vitamin formulation (Blackmores® Executive B Stress Formula) on mood and workplace stress in a sample of full time, healthy employees. The secondary aim is to examine the mechanisms underpinning any mood or workplace stress enhancing actions of B vitamins by examining relationships between cognitive, biological (biochemical, genetic and brain metabolites) and cardiovascular variables over 6 months. By examining the interrelationship between inflammation, cardiovascular health and cognitive performance, the current study aims to identify modifiable risk factors for workplace stress that can be targeted by supplementation. It is hypothesised that 6 month supplementation with Executive B Stress Formula will improve measures of workplace stress, relative to placebo with the greatest effects observed at 6 months. It is also expected that there will be an improvement in general health factors, cognitive performance, biological variables and cardiovascular health. The primary psychological outcome measure is the difference between the B-vitamin groups and placebo group over time on the total stress/strain score of the PSQ from the OSI-R. The primary physiological outcomes will be the differences levels of HCy in blood plasma. The secondary outcome measures are the differences between the B vitamin group and placebo group in mean changes over time on: the other OSI-R Questionnaires and their subscales; the GHQ-12, the state-trait anxiety and mood measured by the Profile of Moods Scale (POMS).

### Centres

The study will be conducted at the Centre for Human Psychopharmacology, Swinburne University, Melbourne. Additional recruitment will occur at NatMed Research Unit, Southern Cross University, NSW.

### Participants

The participant group will consist of 200 full time employees aged between 30–65 years, who report feeling stressed in the workplace. Participants will be randomised to receive Blackmores® Executive B Stress Formula or placebo. Participants will be excluded from the study if they have a psychiatric or neurological disease, significant endocrine, renal, pulmonary, gastrointestinal or cardiovascular disorder, other disorder affecting food metabolism, a diagnosis of diabetes (type 1 or type 2) recent history (past 5 years) of chronic/severe illness (longer than 12 months), current regular alcohol use exceeding 14 standard drinks per week for women or 28 standard drinks per week for men, vision that is not corrected to normal. Participants will also be excluded from the study if they are currently pregnant, breastfeeding or planning to become pregnant in the next 6 months. To be eligible, participants cannot be taking anticoagulant medication (e.g. Warfarin), psychoactive medication including antidepressants, antipsychotics, anxiolytics, illicit drugs or significant cognitive enhancing drugs (e.g. chronic intake of substances such as Gingko). For the neuroimaging component of the study current smokers will not be included. To be eligible on the study day visits, participants must adhere to the study day restrictions. These restrictions include not drinking alcohol for 24 hours prior to the scheduled study day, and not drinking caffeine 12 hours prior to the study day. Participants are also asked to fast for 12 hours prior to the study day appointment so that biochemical markers can be assayed from obtaining fasting blood samples.

The study was ethically approved by the Swinburne University Research Ethics Committee (project number 2012/293) and all participants will provide written informed consent. The trial has been registered with the Australian and New Zealand Clinical Trials Registry (ACTRN12613000294752).

### Procedure

**Figure 1:**
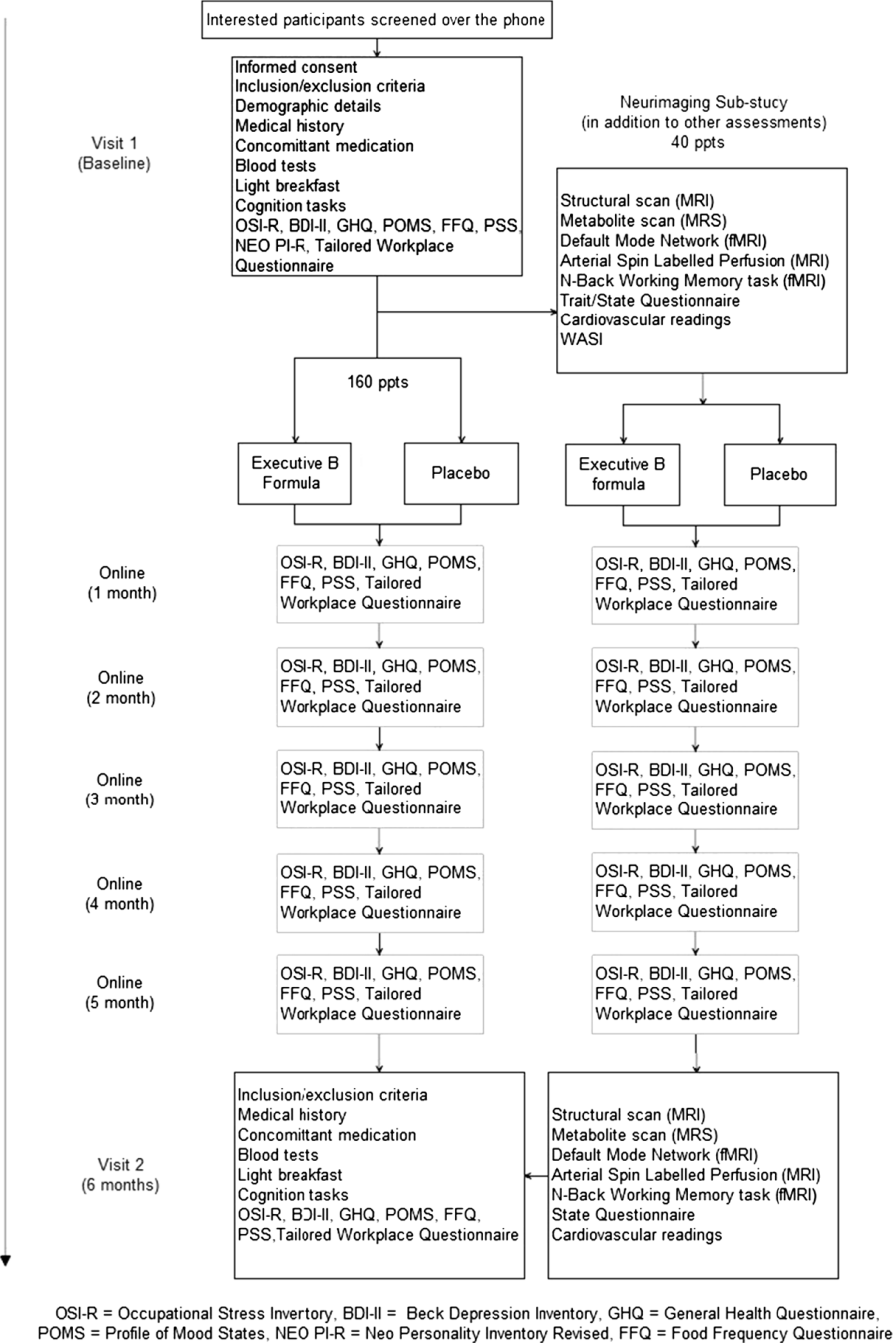
**Protocol flow diagram.**

Eligible participants are required to attend two testing sessions and commit to completing five online questionnaires. An overview of the testing sessions is provided in the clinical trial flow chart (Figure 1). Participants are initially screened over the telephone for eligibility prior to their first visit. During the first visit participants are again screened for eligibility and for compliance to the study day restrictions which were described above. A detailed medical history is also acquired. Voluntary written informed consent is obtained from all participants. Those who are eligible are then asked to give blood and complete a series of workplace stress, personality, mood and general health questionnaires. A light breakfast is provided to participants prior to completing a cognitive test battery. At the conclusion of the session participants are randomised to their treatment group and provided with enough supplements for 6 months. As outlined in Figure 1, participants will have to complete online assessments relating to workplace stress, mood and general health from the first to the fifth month post randomization. At visit two, the 6 month assessment, participants will undergo the same procedure from visit 1. Participants will be asked to return any remaining supplements, enabling the investigators to estimate compliance to treatment by counting the remaining amount.

#### Neuroimaging sub-study

Participants involved in the neuroimaging sub-study will be required for an additional 2 hours on the initial and last visit to the Centre for Human Psychopharmacology (see Figure 1). Additional screening for eligibility for these participants regarding any metallic implants is conducted to ensure their safety to undergo Magnetic Resonance Imaging (MRI) scans. Participants complete a practice version of the *N*-back task being used in this component of the study, to familiarise themselves with the task and to minimise practice effects. Cognitive data obtained during the training session will not be included in statistical analysis. On the first visit both “trait” and “state” assessments of the Spielbeger State-Trait Anxiety Inventory [46] will be administered prior to the MRI scans to determine a baseline anxiety level. The “state” version of the questionnaire will be administered again, after the MRI scan to ensure the experience of the MRI has not impacted on the participant’s responses. This scale is a widely used instrument for measuring fluctuating levels of anxiety. Additionally, participants will complete the vocabulary and matrix reasoning subsets of the Wechsler Abbreviated Scale of Intelligence (WASI) [47] on visit one, which is a reliable measure of general intelligence for use in clinical and research settings. Participants will lie in the MRI scanner whereby different imaging techniques will be employed to determine brain structure, blood flow, brain metabolite levels, resting state connectivity as well as completing a cognitive task (*N*-back) to assess functional connectivity. At the second and final visit, six months post treatment, the same procedure from visit one will be implemented and participants will be asked to complete the “state” version of the STAI before and after the MRI scan.

### Sample size

The sample size of the study is 200 participants, 100 participants in each arm. Power analysis was conducted using G*Power 3.1.2. For a repeated measures design with two groups (treatment vs placebo) and three time points (baseline, three months and six months), previous research conducted at Swinburne University of Technology identified a treatment related improvement of 19% in ratings of psychological distress after three months supplementation with Blackmores® Executive Stress formula.

### Treatments

The Executive B Stress Formulation is manufactured by Blackmores®, Australia. The ingredients and dose of each ingredient of the Executive B Stress Formulation is listed in Table 1. The inert placebo tablets provided by Blackmores® are matched in size and colour to the Executive B tablets and include a small amount of riboflavin (2 mg) so as to provide a similar urine colouration effect. The participants will be instructed to take two tablets, one at breakfast and one at lunch time daily, for the duration of 24 weeks (6 months). To prevent potentially acute supplementation effects, the participants are asked not to take their tablets on the final study visit.

**Table 1:** **Ingredients and dose for the Executive B formulation**

Vitamin B1 (thiamine hydrochloride)	75 mg
Vitamin B2 (riboflavin)	10 mg
Nicotinamide	100 mg
Vitamin B5 (pantothenic acid from calcium pantothenate 75 mg)	68.7 mg
Vitamin B6 (pyridoxine hydrochloride)	25 mg
Vitamin B12 (cyanocobalamin)	30 μg
Vitamin H (biotin)	20 μg
Calcium ascorbate	145 mg
Ascorbic acid (total vitamin C 250 mg)	130 mg
Vitamin E (D-alpha-tocopheryl acid succinate 41.3 mg)	50 IU
Magnesium phosphate	140 mg
Calcium phosphate	100 mg
Potassium phosphate monobasic	117.3 mg
Folic acid	150 μg
*Avena sativa* (Oats) extract equiv. to dry seed	100 mg
*Passiflora incarnata* (Passion flower) extract equiv. to dry herb	250 mg
Lecithin	50 mg
Choline birartrate	25 mg
Inositol	25 mg

### Randomization and safety

Randomization of participants to treatment groups will be determined by random allocation. Each participant number will be assigned to a treatment group (active or placebo) using a computerized random number generator by a disinterested third party ensuring all other parties remain blind to the treatment allocation. Eligible, recruited participants will be assigned a participant number. Participant numbers will be allocated sequentially and once assigned will not be re-used. Randomization codes will be kept in a password protected computer file and will only be accessed in case of emergency. All potential adverse events will be monitored throughout the trial, with oversight from the Swinburne University of Technology Human Research Ethics Committee.

### Primary outcome

The primary study outcome will be the effect of Executive B formula on work related stress as measured by a well validated work related stress measure. The Occupational Stress Inventory-Revised (OSI-R) measures three dimensions of occupational adjustment: occupational stress, psychological strain and coping resources [48]. Within each of these areas a number of subscales provide more detailed information. The instrument yields scores on 14 different scales ranging from “Role Overload” and “Interpersonal Strain” to “Self-Care”. High scores on the subscales measuring “occupational roles” and “personal strain” and low scores on the subscales measuring “personal resources” indicate high psychological stress and poor occupational adjustment. In a previous investigation of occupational stress, this measure identified a reduction in personal strain and depressed mood in response to 90 days of B vitamin supplementation [23].

### Secondary outcomes

A range of cognitive, stress, mood, health, personality, cardiovascular, biochemical, genetic and neuroimaging measures will be collected at different time points as part of the study. These are described below and listed in Table 2.

**Table 2:** **Summary of the secondary outcomes implemented in Executive B across all time points**

	Visit 1 (baseline)	Visit 2 (6 months)
**Screening**	Medical history (ie demographic screen, medication use)	Medical history (ie medication use)
**Biochemical**	MBA & CRP	MBA & CRP
	Homocysteine	Homocysteine
	B Vitamins (B6, B12)	B Vitamins (B6, B12)
	Folate	Folate
	COMT	COMT
	SLC6A4	SLC6A4
	FKBP5	FKBP5
	NPY	NPY
	ADCYAP1R1	ADCYAP1R1
	MTHFR	MTHFR
	CPS1	CPS1
	CBS and DPEP1	CBS and DPEP1
**Health**	FFQ	FFQ
	BDI-II	BDI-II
	POMS	POMS
	GHQ	GHQ
	PSS	PSS
**Cardiovascular**	Brachial BP	Brachial BP
	Aortic BP	Aortic BP
	Carotid-femoral PWV	Carotid-femoral PWV
**Neuroimaging**	Structural MRI	Structural MRI
	Default mode network activation	Default mode network activation
	Metabolite scan (MRS)	Metabolite scan (MRS)
	N-Back working memory (functional MRI)	N-Back working memory (functional MRI)
**Other**	NEO PI-R	Estimate of compliance
	WASI	

COMT = Catechol-O-methyltransferase gene, SLC6A4 = Serotonin-transporter gene-linked polymorphic region, FKBP5 = Glucocorticoid receptor-regulating co-chaperone of stress proteins, NPY = Neuropeptide Y, ADCYAP1R1 = Pituitary adenylate cyclase-activating polypeptide receptor, MTHFR = Methylenetetrahydrofolate reductase, CPSI = Carbamoyl phosphate synthetase 1, CBS and DPEP1 = Cystathionine –β-synthase and Dipeptidase 1, FFQ = Food Frequency Questionnaire, BDI-II = Beck Depression Inventory II, POMS = Profile of Mood States, GHQ = General Health Questionnaire, PSS = Perceived Stress Scale, NEO PI-R = NEO-Personality Inventory Revised, WASI = Wechsler Abbreviated Scale of Intelligence.

#### Cognitive

To investigate the effect of supplementation on cognitive performance the Swinburne University Computerised Cognitive Assessment Battery (SUCCAB) will be implemented at visit one (baseline) and 6 months post supplementation. The SUCCAB is a validated battery of 8 tests assessing a range of cognitive functions that decline with age [49]. Cognitive enhancing effects of multivitamin supplementation in middle aged [50] and elderly individuals [51] have been identified using this cognitive battery of tasks.

#### Stress, mood, health and dietary habits

Several self-report questionnaires will be used to assess stress, mood and general health. Symptoms of stress will be measured with the Perceived Stress Scale (PSS) [52]. Depressive symptoms and state-trait anxiety will be measured with the Beck Depression Inventory II (BDI-II) [53] and the Spielberger State-Trait Anxiety Inventory [46] respectively. Further assessment of mood will be conducted with the Profile of Mood States [54]. Pre-existing general health and general health for the duration of the trial will be assessed with the General Health Questionnaire (GHQ) [55]. Dietary habits will be inferred from an in-house Food Frequency Questionnaire.

#### Personality

Participants will be asked to complete the NEO PI-R [56]. The NEO PI-R is the most widely used questionnaire used to assess personality traits. Consisting of 240 items, this questionnaire assesses the Five Factor Model of personality by scoring participants across personality dimensions and related facets of Openness, Conscientiousness, Extroversion, Agreeableness and Neuroticism. As personality traits are considered stable across time, participants will only complete this questionnaire once at the beginning of the trial. The research investigators will examine how different personality types respond to the intervention.

#### Cardiovascular

The results of a meta-analysis predicted that occupational stress increases the risk of heart disease by 50% [57]. Additionally, evidence suggests that a contributing factor in cognitive decline is an increase in arterial stiffness with ageing [58]. Given the interrelationship identified between B group vitamins and HCy with cognition and cardiovascular health, it could be argued that chronic supplementation of B vitamins may have a positive influence on cardiovascular function. Therefore, cardiovascular measures in the current study will enable exploration of whether improvements to cardiovascular function are a mechanism by which B group vitamins improve cognitive ability.

Brachial blood pressure measuring standard brachial systolic and diastolic pressure alone with a clinically validated automated sphygmomanometer may not be a true assessment of central aortic events [59]. Including measurements of aortic pressures (PWA: Pulse Wave Analysis; central aortic pressures) and carotid-femoral Pulse Wave Velocity (PWV; aspects of arterial stiffness) provides a reliable measurement of cardiovascular events [60]. Measuring PWV between the carotid and femoral artery sites is considered a gold standard in the assessment of artery stiffness [61].

PWA to measure participant’s central pulse pressure, augmented pressure and augmentation index will be obtained using a SphygmoCor® device (Model EM4C, AtCor Medical, Sydney, Australia). Participants will be supine and have an arm cuff placed around their brachial artery on their left side with measurements obtained after 10 minutes of rest. To ensure the accuracy of the assessment, blood pressure will be taken three times, the first reading will be discarded and the second two will be averaged.

The same device will be used with participants to measure PWV between the carotid and femoral arteries to obtain measurements of arterial stiffness. The averaged brachial systolic and diastolic pressures from the first assessment will be entered into the computer software (Version 1.2.0.7) to assist in measuring arterial stiffness. The distance between the carotid and femoral artery and femoral artery to cuff sites will be measured using a flexible tape measure and entered into the software program whereby the length of the aorta is estimated by the computer program. A femoral cuff will be placed over the participant’s femoral artery and a tonometer pressure sensor applanating the carotid artery, to capture the carotid waveform, will be applied to obtain measurements of aortic stiffness.

#### Biochemical

Blood sampling is to be conducted via venepuncture by a registered nurse or qualified phlebotomist on each of the testing days, (visit 1 and visit 2) following a period of fasting from the night before. Biochemical markers of Vitamin B12, Vitamin B 6, Folate and HCy will be obtained. Additionally, safety profiling will be measured through a full blood count, high sensitivity C-reactive Protein and biochemical Liver Function Tests.

#### Genetic

Blood samples via venepuncture will be used to provide specific genetic information which has been shown to be implicated in stress processing. In a review of current genetic work, five candidate gene polymorphisms were identified as being illustrative of individual differences in emotion processing [62]. They are: catechol-O-methyltransferase (COMT), serotonin transporter (SLC6A4), neuropeptide Y (NPY), a glucocorticoid receptor-regulating co-chaperone of stress proteins (FKBP5) and pituitary adenylate cyclase-activating polypeptide receptor (ADCYAP1R1). Emotional processing could be implicated in stress processing, and any change in expression due to vitamin supplementation. Processing of emotions is critical to most aspects of human behaviour, and individual differences in the processing of emotional stimuli exist as functions of personality (Neuroticism), neuropsychiatric disorders, and with specific reference to this proposal, workplace stress.

#### Neuroimaging

Neuroimaging will be conducted in a subset of 40 participants in order to explore the *in vivo* mechanisms of action of Executive B formula in the brain to elucidate underlying cognitive and health effects of B group vitamins. Previous imaging studies looking at vitamin supplementation have been conducted using electroencephalography (EEG; e.g., Macpherson H, Ellis KA, Sali A and Pipingas A [51]). Few studies have investigated the *in vivo* effects of supplements on brain metabolites as measured with magnetic resonance spectroscopy (MRS) and functional magnetic resonance imaging (fMRI). The technique of MRS has been used to study alterations in brain metabolites of people with clinical disorders like depression [63] and schizophrenia [64], or Alzheimer’s disease and mild cognitive impairment [65, 66]. However, as far as we understand, no neuroimaging studies have been conducted using the technique of MRS to investigate the relationship of brain metabolic function, cognition and cardiovascular health in response to B vitamin supplementation.

Structural and functional MRI (fMRI) scans will be acquired using a Siemens 3 Tesla Tim Trio MRI scanner (Erlargen, Germany), located at the Centre for Human Psychopharmacology, Swinburne University of Technology. A structural scan will be obtained of each participant and used as a reference point for further functional scans. Scanning in a resting state will occur in order to assess activity in the default mode network (DMN) for approximately 6 minutes. Following DMN there will be scanning for brain metabolite levels in three regions of interest using the technique of MRS for approximately 35 minutes. Following MRS an arterial spin labelled perfusion sequence will be conducted for approximately 6 minutes to investigate the brain’s tissue perfusion. Finally, changes in the blood oxygenation-level dependent (BOLD) signal will be analysed while participants compete an in-scanner version of the *N-*Back working memory task (approximately 10 minutes) developed for The Human Connectome Project [67].

### Analysis

The primary analysis will investigate the effect of treatment on workplace stress levels over the course of the study using Repeated Measures Analysis of Variance (ANOVA) techniques (with linear mixed modelling used to account for any missing data). Secondary outcome variables will be analysed using similar statistical techniques. Pearson’s correlation coefficients or the non-parametric equivalent, Spearman’s R will be used to investigate relationships between other variables of interest such as stress, biochemical, cardiovascular or mood/health factors collected at baseline. Results will be considered statistically significant at an alpha level of *P* < 0.05 corrected for multiple comparisons.

Analysis of brain metabolites from MRS as well as the fMRI data during *N*-Back working memory task will be conducted using a region of interest (ROI) approach. Between group (Executive B Formula versus inert placebo) functional differences in predefined brain regions will be statistically analysed. The ROIs that will be analysed in the *N*-Back working memory task will include the dorsolateral, ventrolateral and medial prefrontal cortex, anterior cingulate, parietal cortex and sensorimotor cortex [68]. The MRS ROIs that will be analysed will include the dorsolateral prefrontal cortex, posterior cingulate, and amygdala for their well-established association with stress and cognitive factors [65, 69, 70].

## Conclusions

Occupational stress is a multibillion dollar problem. Effective strategies reducing occupational stress are urgently required. Dietary supplementation with B group vitamins may be an economically viable and sustainable intervention. Reducing occupational stress will have enormous benefits for decreasing stress claims, absenteeism, and increasing work productivity. Vitamin B supplementation is also likely to have additional health and quality of life benefits.

### Trial status

The trial is currently recruiting.

## Abbreviations

ANOVA: Analysis of Variance
ANZCTR: Australia and New Zealand Clinical Trials Registry
BDI-II: Beck Depression Inventory II
BOLD: Blood oxygenation level dependent
BP: Blood pressure
DMN: Default Mode Network
EEG: Electroencephalography
Exec B: Executive B Study
FFQ: Food frequency questionnaire
fMRI: Functional magnetic resonance imaging
GHQ: General health questionnaire
HCy: Homocysteine
MRS: Magnetic resonance spectroscopy
ROI: Region of Interest
NEO PI-R: NEO personality-inventory revised
OSI-R: Occupational stress inventory-revised
POMS: Profile of Mood States
PSS: Perceived stress scale
SAM: S-adenosylmethionine, SUCCAB, Swinburne University Computerized Cognitive Assessment Battery
WASI: Wechsler abbreviated scale of intelligence.
